# Emerging cancer disease burden in a rural sub-Saharan African population: northeast Nigeria in focus

**DOI:** 10.3389/fonc.2024.1380615

**Published:** 2024-07-17

**Authors:** Uchenna S. Ezenkwa, Aliyu Ibrahim Lawan, Musa Abubakar Garbati, Dauda E. Suleiman, Dauda A. Katagum, Abba Kabir, Adamu Isa Adamu, Abubakar Kolomi Modu, Olaniyi David Olanrewaju, Rufai Abdu Dachi, Yusuf Mohammed Abdullahi, Muhammed Alkali, Danladi Adamu Bojude, Hadiza Abdullahi Usman, Ayodele Omotoso, Matthew Schlumbrecht, Sophia H. L. George, Bala Mohammed Audu

**Affiliations:** ^1^ Department of Histopathology, Federal University of Health Sciences Azare, Azare, Bauchi, Nigeria; ^2^ Department of Histopathology, College of Medical Sciences, Gombe State University, Gombe, Gombe, Nigeria; ^3^ Directorate of Research, Innovation and Development, Federal University of Health Sciences Azare, Azare, Bauchi, Nigeria; ^4^ Department of Histopathology, College of Medical Sciences, Abubakar Tafawa Balewa University, Bauchi, Bauchi, Nigeria; ^5^ Department of Obstetrics and Gynaecology, Federal University of Health Sciences Azare, Azare, Bauchi, Nigeria; ^6^ Department of Histopathology, College of Medical Sciences, University of Maiduguri, Maiduguri, Borno, Nigeria; ^7^ Department of Histopathology, Yobe State University, Damaturu, Yobe, Nigeria; ^8^ Department of Pathology, Federal Medical Centre Nguru, Nguru, Yobe, Nigeria; ^9^ Department of Haematology and Blood Transfusion, Federal University of Health Sciences, Azare, Nigeria; ^10^ Department of Haematology, Abubakar Tafawa Balewa University, Bauchi, Bauchi, Nigeria; ^11^ Department of Medicine, Federal University of Health Sciences, Azare, Bauchi, Nigeria; ^12^ Community Oncology and Epidemiology, Gombe State University, Gombe, Gombe, Nigeria; ^13^ Department of Obstetrics and Gynecology, Federal Medical Centre Nguru, Nguru, Yobe, Nigeria; ^14^ Division of Gynecologic Oncology, Department of Obstetrics, Gynaecology and Reproductive Sciences, Sylvester Comprehensive Cancer Centre, University of Miami Miller School of Medicine, Miami, FL, United States

**Keywords:** cancer burden, age-specific rate, northeast Nigeria, sub-Saharan Africa, cancer care disparity

## Abstract

**Introduction:**

Sub-Saharan Africa (SSA) is plagued by myriads of diseases, mostly infectious; but cancer disease burden is rising among non-communicable diseases. Nigeria has a high burden of cancer, however its remote underserved culturally-conserved populations have been understudied, a gap this study sought to fill.

**Methods:**

This was a cross-sectional multi-institutional descriptive study of histologically diagnosed cancers over a four-year period (January 2019-December 2022) archived in the Departments of Pathology and Cancer Registries of six tertiary hospitals in the northeast of Nigeria. Data obtained included age at diagnosis, gender, tumor site and available cancer care infrastructure. Population data of the study region and its demographics was obtained from the National Population Commission and used to calculate incident rates for the population studied.

**Results:**

A total of 4,681 incident cancer cases from 2,770 females and 1,911 males were identified. The median age at diagnosis for females was 45 years (range 1–95yrs), and 56 years (range 1–99yrs) for males. Observed age-specific incidence rates (ASR) increased steadily for both genders reaching peaks in the age group 80 years and above with the highest ASR seen among males (321/100,000 persons) compared to females (215.5/100,000 persons). Breast, cervical, prostatic, colorectal and skin cancers were the five most common incident cancers. In females, breast, cervical, skin, ovarian and colorectal cancers were the top five malignancies; while prostate, haematolymphoid, skin, colorectal and urinary bladder cancers predominated in men.

**Conclusion:**

Remote SSA communities are witnessing rising cancer disease burden. Proactive control programs inclusive of advocacy, vaccination, screening, and improved diagnostics are needed.

## Introduction

Cancer is predicted to cause a mortality of about one million by 2030 in Africa (1). The continent was estimated to have contributed about 5.7% (1,100,100) incident and 7.2% (712,800) mortality rates to the global cancer burden in the year 2020 (2). In sub-Saharan Africa (SSA), cancer as a disease is expected to rise by about 85% based only on changing demographics characterized by population growth (3). Added to the lack of effective health care institutions, this will spell catastrophe for the continent in general, and Nigeria in particular, as Nigeria has a high cancer disease burden, ranking second after Egypt in Africa, and first in SSA (4). Additionally, the upward trend in globalization facilitated by increasing affordability of digital devices, has allowed an exchange of cultural and lifestyle preferences, some of which are cancer-promoting (such as dietary habits, smoking, and alcohol indulgences). These factors are easily adaptable to local communities (5–7).

The present generation of children will likely experience further increases in cancer cases in the near future if mitigating strategies are not initiated now. Indeed, it is estimated that low resource countries of the world will witness a greater increase in cancer incidence and mortality by 2040 (2). The state of unpreparedness of these economically disadvantaged countries is worsened by a lack of reliable cancer statistics to drive health system planning (8). Efforts in this direction have begun in Nigeria with the establishment of the Cancer Registry Network; however, not much of the population is covered by the participating registries, with only a few being Population-based (9, 10). Data from North-eastern Nigeria are far from being harmonized and instructive as most have been on individual cancer site-specific case descriptions. This present study was therefore conceived to address this knowledge gap by piecing together information on confirmed cancer cases and presenting them as a unified report from most parts of the zone. It is also hoped that the findings will suggest areas of priority attention as a foundation for future forecasts and intervention planning.

## Materials and methods

Study Design: This is a cross-sectional retrospective descriptive study of histologically confirmed malignancies in the tertiary hospitals in the Northeast of Nigeria over a four-year period (January 2019 to December 2022). De-identified archived histopathology reports in the Departments of Pathology and cancer registries data (where available) of the participating institutions were reviewed and data on cancer cases were retrieved together with information on age at diagnosis, gender, and tumor site. Also obtained was information on the available cancer infrastructure in each of the institutions to ascertain the cancer control capacity in the region. The retrieved information was double checked for clarity and validity by the collaborating Pathologists (USE, AIL, DES, AK, AIA, and MAK) prior to further analysis.

**Figure 1:**
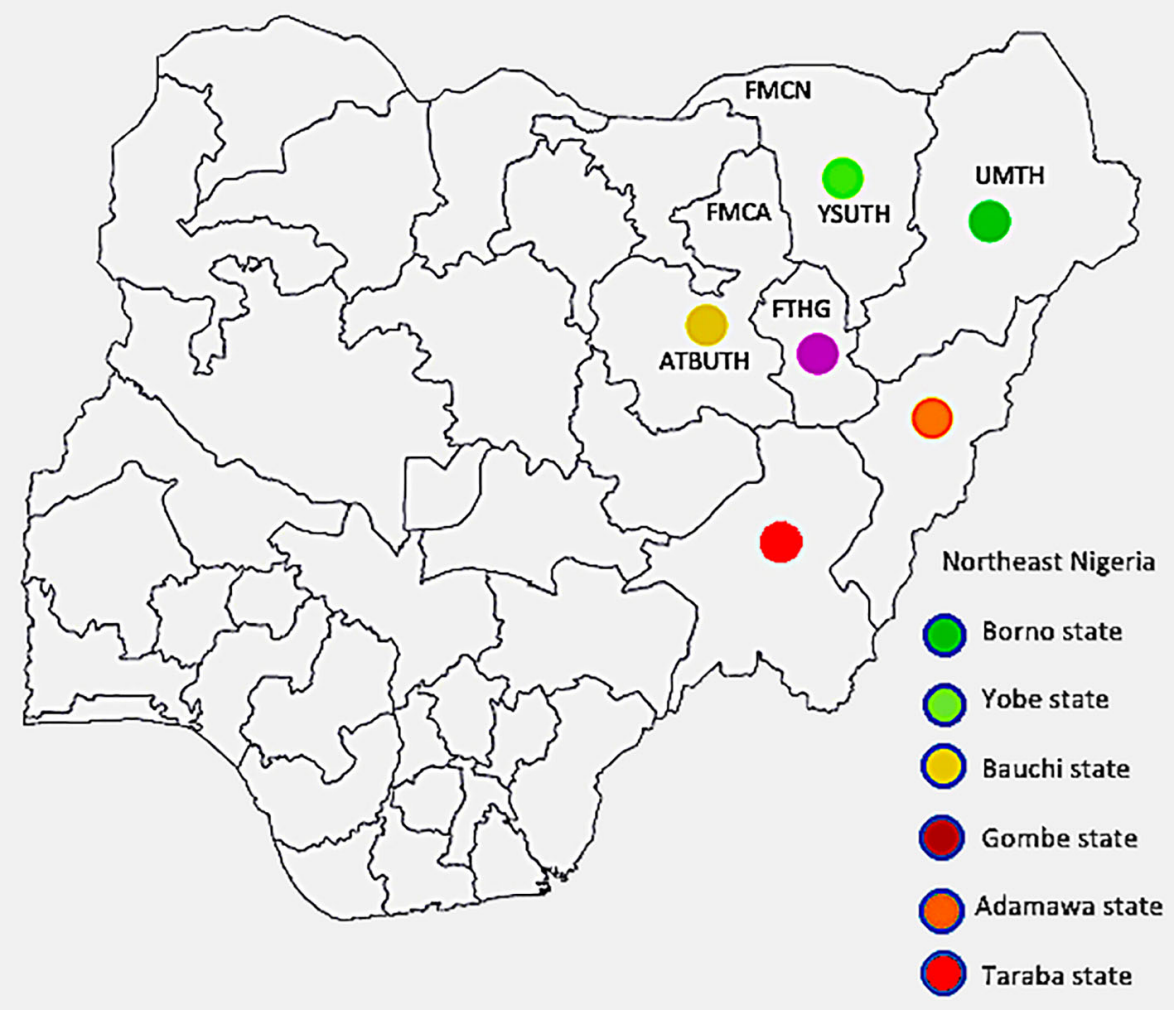
Map of Nigeria with the northeast states highlighted The institutions from which data was obtained are indicated by their abbreviations (FMCN, Federal Medical Centre Nguru; YSUTh, Yobe State University Teaching Hospital; UMTH, University of Maiduguri Teaching Hospital; FTHG, Federal Teaching Hospital Gombe; FMCA, Federal Medical Centre Azare; and ATBUTH, Abubakar Tafawa Balewa University Teaching Hospital), with Bauchi and Yobe states each having two tertiary hospitals, while Borno and Gombe have one hospital each. The position each institution is placed is only a rough approximation of its location within the respective states.

Geographical area of the study: Northeast Nigeria stretches from latitudes 6 28" N and 13 44" N to longitude 8 44" E and 14 38" E. It consists of six states namely, Adamawa, Bauchi, Borno, Gombe, Taraba and Yobe as depicted in **Figure 1**. As at the 2006 census, the region’s total population was put at 18, 984,299 or 13.52% of the total population of Nigeria, covering a landmass of about 272,395km^2^, which is 29.45% of Nigeria’s total landmass (11). With a projected annual growth rate averaging about 2.5% per annum, the population was projected to rise to 30,541,872 in 2022 (12).

Participating institutions: Six tertiary hospitals in four out of the six states in the northeast zone of Nigeria contributed data for this study. They are Abubakar Tafawa Balewa University Teaching Hospital (ATBUTH), Bauchi and Federal Medical Centre Azare (FMCA), both in Bauchi State; University of Maiduguri Teaching Hospital (UMTH), Borno State**;** Federal Teaching Hospital Gombe (FTHG), Gombe State; Federal Medical Centre Nguru (FMCN), and Yobe State University Teaching Hospital (YSUTH), both in Yobe State (**Figure 1** above). These institutions serve as referral centers for the secondary and primary health care facilities in the respective states and are strategically located to ensure accessibility to the populace.

Population distribution data by age and sex for the participating states were extracted from the Nigeria National Population Commission data of 2020 which incorporated projections for the year 2022, thus encompassing the study period (12).

Data analysis: Descriptive (frequency) statistics analysis was done using the statistical package for social sciences (IBM SPSS software) version 20. The output was categorized into cancer sites by proportions and presented as texts, tables and figures. Age-specific rates (ASR) were calculated for each cancer type, using the 2022 estimated population figures. 95% confidence intervals were generated. Other malignancies with incidence rates less than 1% were aggregated together and classified as “others” for clarity of presentation.

## Results

### Demographic characteristics of study sites

The four participating states of the northeast zone have a combined population of 22,029,974 with 50.4% of them males. Bauchi state has the highest population density followed by Borno state (**Supplementary Table 1**). Persons younger than 50 years constituted 91.5% of the entire populace, and among these, individuals aged less than 30 years accounted for about 80% (**Supplementary Table 2**).

### Cancer demographics

**Figure 2:**
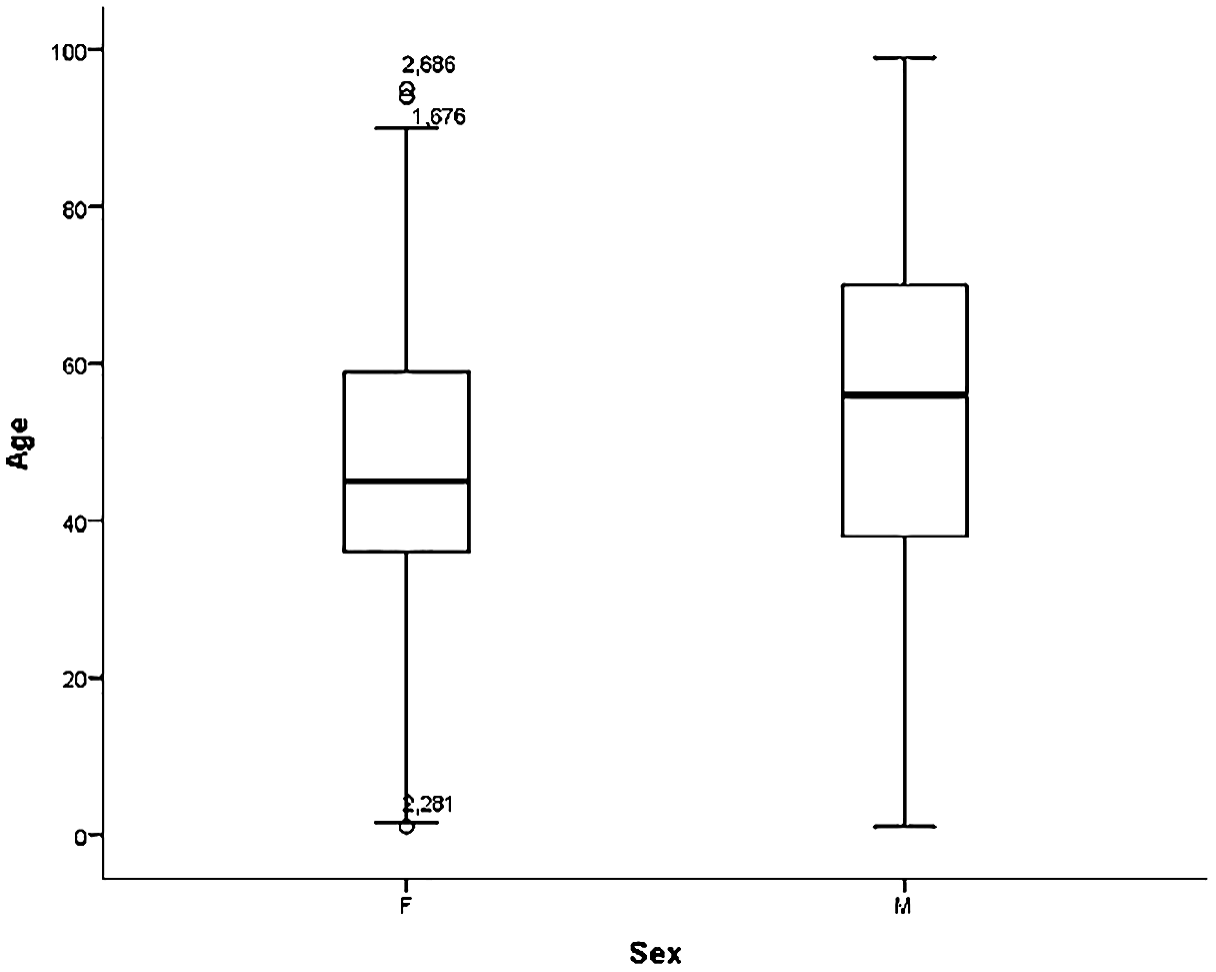
Age distribution in years of females and males diagnosed with cancer. F, Females; M, Males.

Case contribution by institution was highest in FTHG with 1,812 cases, followed by UMTH (1,071), ATBUTH (958), FMCA (314), FMCN (290) and YSUTH (256), giving a total of 4,701. Of these, 20 entries were adjudged inconsistent with cancer diagnosis and were excluded. Among the 4,681 cancer cases included in this study, 69% (2,770) of them were females giving a female-to-male ratio of 1.5:1. The median age at diagnosis for the entire cohort was 50 years (range 1–99 years, interquartile range 36 years), the 25^th^ and 75^th^ percentiles of the age being 25 and 61 years respectively. Females presented on average a decade earlier than males (45 years vs 56 years) as shown in **Figure 2**, but with a narrow difference in age at presentation in the first quartile for both sexes (36 years for females and 38 years for males). This gap broadened again at the third quartile (59 years for females; 70 years for males).

Grouping the age demographics by decades, an increase in cancer incidence was observed with increasing age. About 7.5% (349/4681) of the cases were recorded among children and adolescents while 15.5% (725/4681) occurred in the more elderly individuals (70–99 years). There was a subtle plateau in occurrence of cases between the fifth and seventh decades of life with a minimal drop in the number of cases as the years went by. Fifty-one (1.1%) of the cases had no documented age.

**Figure 3:**
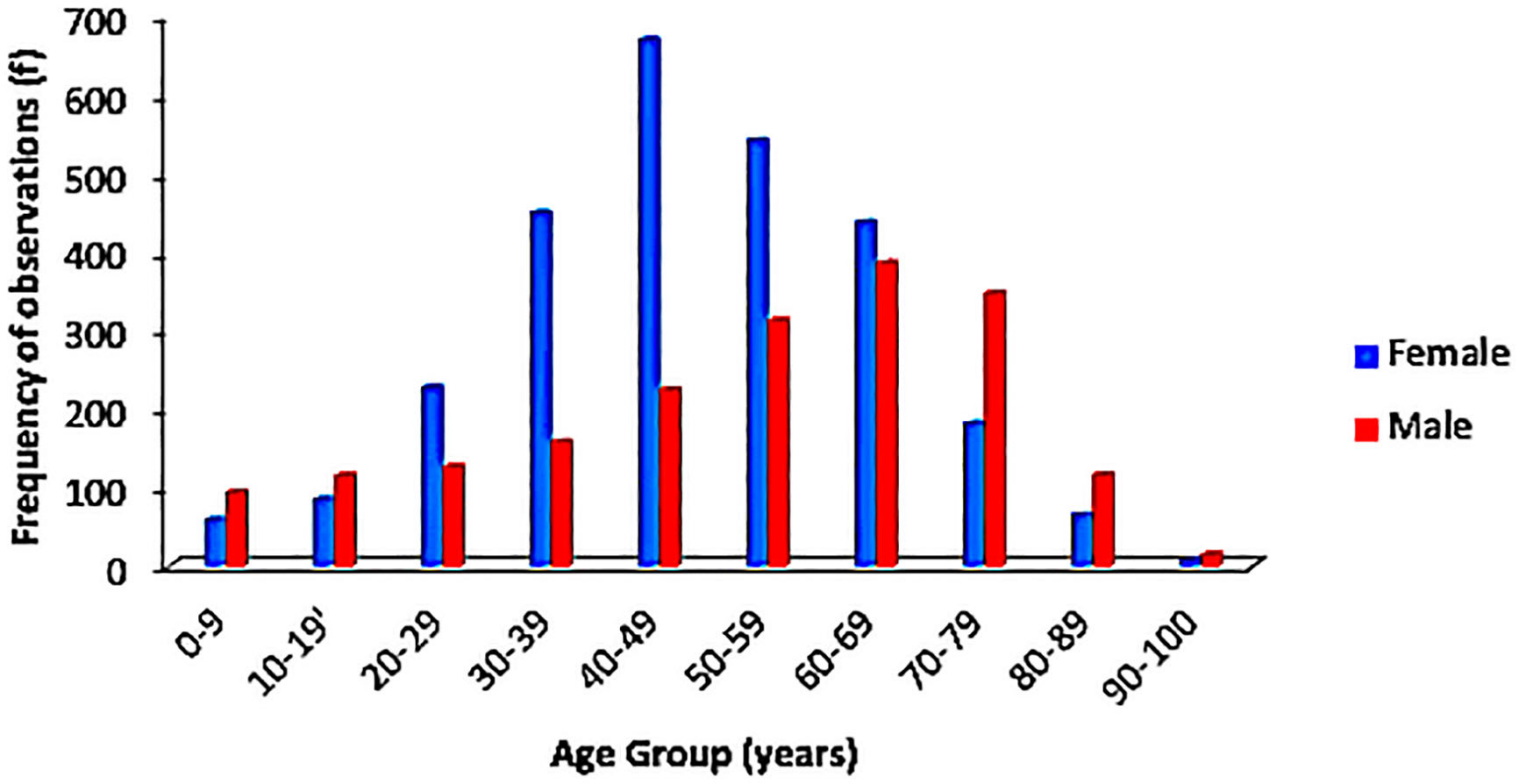
Age distribution comparing females and males by decades as observed in the population.

Gender-matched age distribution is displayed in **Figure 3**. Overall, males had a more sustained rise in number of cases across all age groups, while females had a somewhat bell-shaped distribution. This is influenced by the differences in the peak age group for both genders; the modal age for females was 40–49 years while the males at 60–69 years, although both tailed off at their extremes of age.

**Figure 4:**
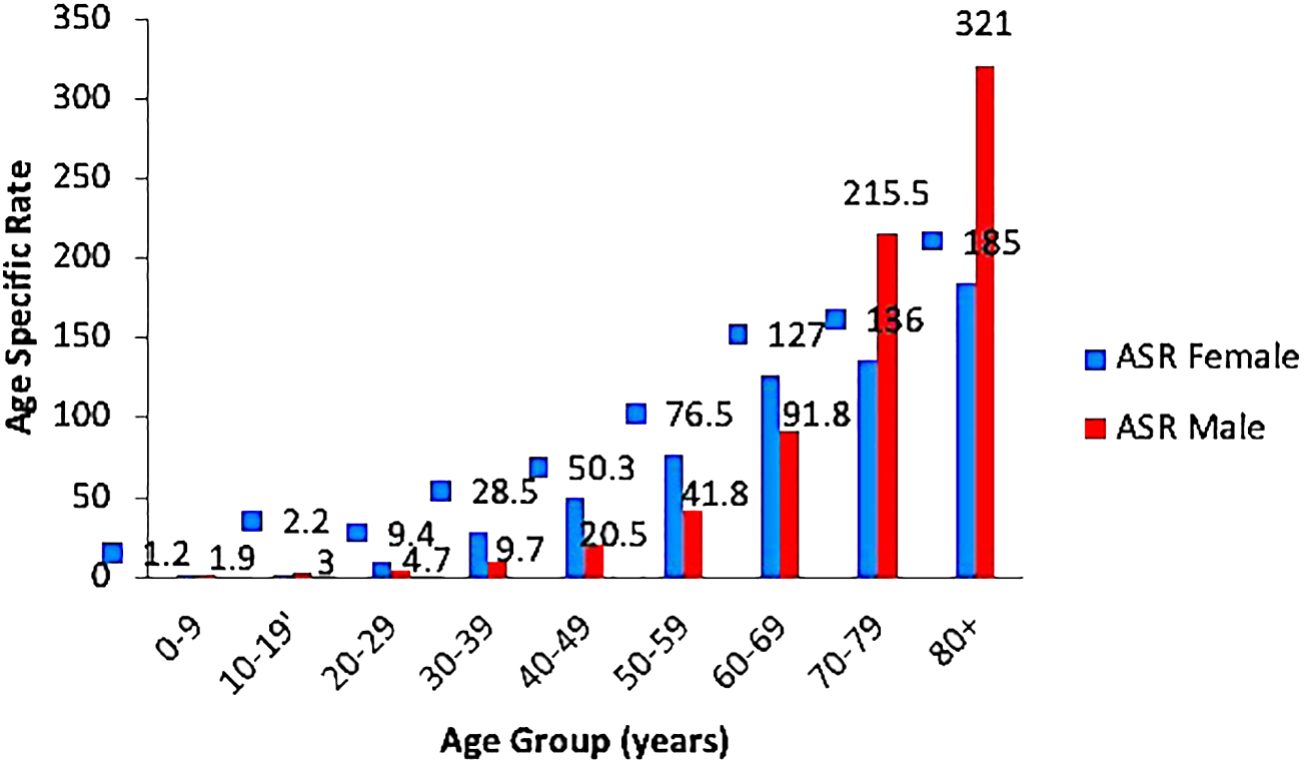
Age-specific rates for both females and males.

Crude ASR for all sites and genders was 15.9/100,000 persons; and 12.4 and 18.3, for males and females, respectively. Further segregation according to gender revealed that the ASR increased steadily in both genders reaching a peak in those aged 80 years and older. The highest ASR of 321 per 100,000 persons was seen among males (**Figure 4**).

Cancer distribution by disease site is presented in **Table 1**, in the order of decreasing frequency.

**Table 1:** Cancer incidence by anatomical sites.

Cancer Site	Frequency (f)	Percentage (%)
**Breast**	1072	22.9
**Cervix**	576	12.3
**Prostate**	483	10.3
**Colorectal (including Anorectum)**	324	6.9
**Skin**	312	6.7
**Haematolymphoid**	284	6.1
**Bone and soft tissues**	190	4.1
**Bladder**	159	3.4
**Ovary**	128	2.7
**Liver**	124	2.6
**Uterus**	112	2.4
**Stomach**	109	2.3
**Kidney**	78	1.7
**Nasopharynx**	59	1.3
**Ocular**	63	1.3
**Others**	429	9.2
**Tumor site missing**	179	3.8
**Total**	**4,681**	**100**

Breast cancer ranked highest among the cases followed by cancer of the uterine cervix and then prostate cancer. Renal, nasopharyngeal, and ocular cancers were among the least common sites involved.**Table 2** shows the incidence by gender and median age of each cancer type. Assumed age-standardized incidence rate (ASR) is also calculated for these tumors. Low ASR for some adult cancer types is noted when such cancers occur in children or early adolescent age as seen for colorectal cancer. Besides the gender related malignancies, there was consistent male preponderance for every cancer type.

**Table 2:** Cancer distribution by anatomical site, age, gender, and age specific rates.

Site	Number	Age (years)	Sex		ASR (95% CI)
		Median(range)	Male	Female	
**Breast**	1072	45 (16–90)	37	1035	7.9 (7.4-8.4)
**Cervix**	576	50 (20-94)	–	576	8.7 (8.0-9.4)
**Prostate**	483	70 (32-98)	483	–	11.9 (10.8-13.0)
**Colorectal**	324	50 (20-92)	180	144	2.4 (2.1-2.7)
**Skin**	312	50 (3-93)	177	135	1.0 (0.9-1.1)
**Haematolymphoid**	284	37 (3-90)	187	97	0.9 (0.8-1.0)
**Bone and Soft Tissue**	190	25 (1-85)	118	72	0.6 (0.5-0.7)
**Bladder**	159	57.5 (11-87)	126	33	0.8 (-0.8-2.4)
**Ovary**	128	48.5 (8-78)	–	128	1.2 (1.1-1.3)
**Liver**	124	48 (2-85)	87	37	0.4 (0.3-0.5)
**Uterus**	112	55 (20-95)	–	112	1.7 (1.4-2.0)
**Stomach**	109	57 (25-85)	60	49	1.0 (0.8-1.2)
**Kidney**	78	9 (1-88)	44	34	0.3 (0.2-0.4)
**Ocular**	62	14 (1-97)	40	22	0.2 (0.1-0.3)
**Nasopharynx**	59	39 (2-72)	36	23	0.2 (0.1-0.3)
**Total**	**4072**		**1,575**	**2,497**	

Numbers presented here represents only cases with documented anatomic sites.

**Figure 5:**
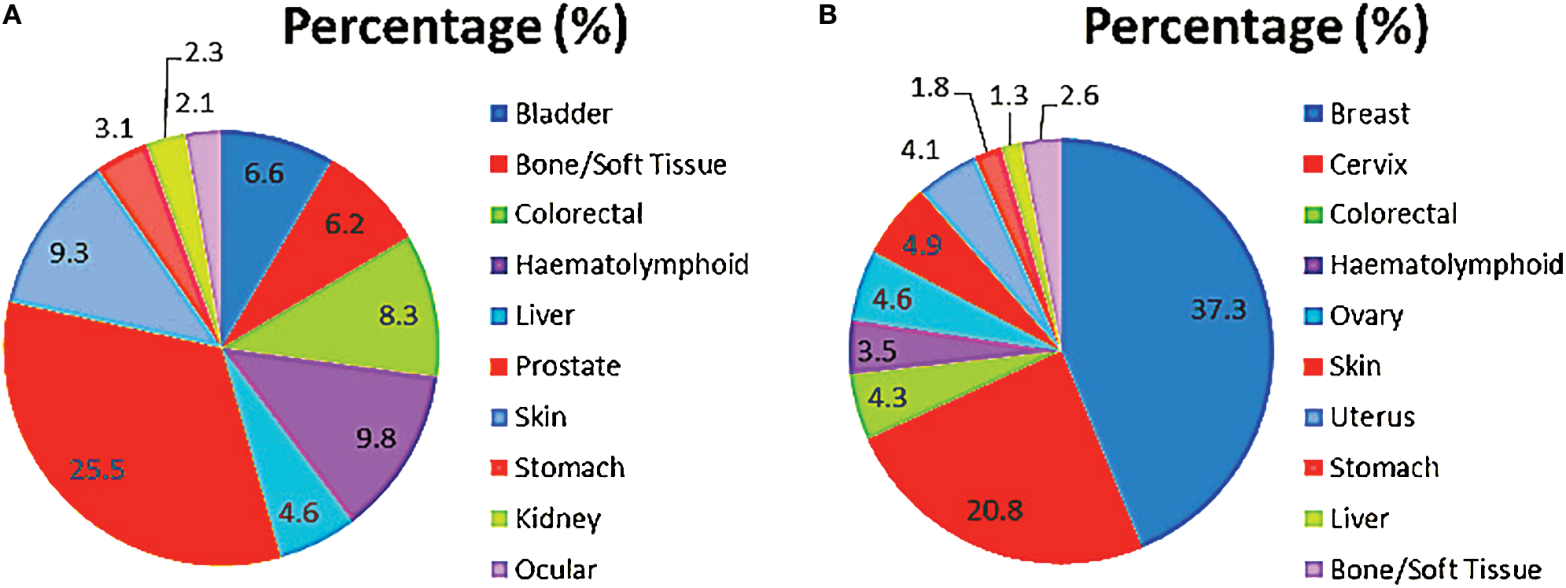
Top 10 cancers in males **(A)** and females **(B)**.

In males, prostate cancer was the most common, accounting for about one-fourth of all cancers, and followed by haematolymphoid malignancies, skin, colorectal and bladder cancers in that order (**Figure 5A**). These cases accounted for greater than three-fifths (77.5%; 1481) of all incident male cancers.

Among the females, breast, cervix, skin, ovary and uterus were the most common cancer sites. Breast and cervical cancers accounted for over half of all incident cases overall (**Figure 5B**). These top 10 female cancers (breast, cervix, colorectal, haematolymphoid, ovary, skin, uterus, stomach, liver, bone/soft tissue) account for over four-fifths (85.3%; 2362) of female cancers during the period under review.

### Cancer care infrastructure

Two out of the four teaching hospitals (FTHG and UMTH) had at the time of collection of data one brachytherapy and 3 radiotherapy machines. None had a positron emission tomography (PET) machine. There were 2 radiotherapists, one medical physicist, one surgical oncologist, one medical oncologist, and one pediatric oncologist serving the entire 6 states of northeast Nigeria with a population of about 30 million people. None of the institutions had a palliative care service in place.

## Discussion

The observed frequency of cancer cases of 4,681 in the studied location is high considering the fact that the region is predominantly a rural and semi-urban agrarian population with low per capita income (13). A decade earlier, Jedy-Agba et al. reported a similar frequency of 4,521 from two large population-based cancer registries in Nigeria, namely, Ibadan in Southwest, and Abuja in North central of Nigeria (14). Credence to this is that ASR in our adult population approximates slightly with those found in Ibadan and Abuja. Although the authors only reported their findings over a two-year period (2009–2010), their studied population was also smaller numbering about 7,922,435 as at year 2010 compared to the present study population (Bauchi, Borno, Gombe and Yobe states) of 22,029,974 as at year 2022 (12) which might downplay the size effect of this disease in the index locality (12). However, the use of hospital-based registry data in this study strongly suggests under-reporting of cases as opposed to population-based registry data in Ibadan and Abuja. Furthermore, it needs to be emphasized that Abuja, Nigeria’s capital city, and Ibadan, a cosmopolitan city in Nigeria’s economic west, are likely experiencing remarkable higher impact of urbanization, westernization and globalization, and these are known risk factors driving cancer incidence in countries with high human development indices (2, 3, 14). Recording a similar figure in the present study suggests a need for more research to understand the determinants of cancer diseases in our environment and similar sub-Saharan Africa communities.

Cancer spectrum in this study is somewhat similar with national, regional (Africa) and global picture except for little differences. Worldwide, breast (11.7%), lung (11.4%), colorectal (10.0%), prostate (7.3%) and gastric (5.3%) cancers were the top five malignancies in 2020, breast cancer dominating in the present study by nearly double (22.9%) its global proportion (2). In Asia, lung cancer was the commonest (13.8%), followed by breast (10.8%), colorectal (10.6%), stomach (8.9%) and liver cancers (6.9%); while in Europe, breast (13.1%), colorectal (12.9%), lung (11.8%) and prostate (11.7%) cancers were the top four cancers in that order, breast cancer proportion in our study again doubling those in these studies (15, 16). Except for the absence of liver cancer in the top five cancers in the present study, we found a striking similarity with data in the African continent which had breast (16.8%), cervical (10.6%), prostate (8.4%), liver (6.4%) and colorectal (6.0%) cancers as the top five cancers (4). Likewise, population-based registries in Nigeria also reported breast, cervical and prostate cancers as the commonest in the country, similar to our finding (14).

While lung and liver cancers featured prominently among the top incident malignancies globally, lung cancer did not feature in the top 10 in the present study, while liver cancer ranked 10^th^. Whereas the effect of smoking as a cause of lung cancer requires long duration to manifest, hepatitis virus infection in Africa often occurs very early, with mother-to-child transmission assuming a worrisome proportion as a cause of liver cancer (7, 17). Thus, younger age and reduced life-expectancy in the continent may support lower rate of lung cancer in our environment, in addition to the effect of under-diagnosis arising from lack of skilled personnel and diagnostic facilities (7, 18, 19). On the other hand, lower liver cancer cases could be due to non-documentation in the Pathology departments because most hepatologists presently avoid liver biopsy, rather relying on clinical features, serum alpha-fetoprotein levels and radiological imaging for diagnosis. Considering the influence of missed cases on policy making about the disease, it is therefore expedient to expand data gathering across the zone and indeed other similar practice settings to include clinically diagnosed cases in order to reasonably study the epidemiology of these cancers and accommodate them in the control and response strategy design (19).

There is similarity in the gender predilection of cancers in this study and others from Nigeria and Africa among women (4, 14). In contrast, global and United States estimates are dominated by male preponderance (2, 20). This difference is driven by the high prevalence of female breast and cervical cancers in Africa. The high incidence of female breast cancer follows a varying pattern of risk factors globally. For example, while screening mammography, older age, fewer births with associated lower breastfeeding practice, obesity, alcohol intake and younger age at menarche characterize the high economic index societies, these factors are less evident in low and middle income countries of SSA (21). Rather, the trend observed in Africa could be accounted for by rapid population growth (22). As the economic fortunes of SSA improves, higher earning may lead to a rise in all of the aforementioned risk factors with sustained high incidence rates of breast cancer (7, 21, 23).

Concerning cervical cancer, the determinants are skewed against African countries due to the persistent high incidence rate of cervical cancer driven by ignorance about the disease and low health literacy generally, as well as lack of institutionalized screening programs (3, 24, 25). Studies in Nigeria have shown that awareness and knowledge about cervical cancer and its preventive strategies could go as low as 8% in some population with uptake of screening ranging from 1.5% to 8.0% (26–28). As has been demonstrated elsewhere, for example in the United States, cervical cancer incidence can decline if public health policy such as advocacy and vaccination against the human papilloma virus is implemented at the population level (20). This is urgently needed in low income communities such as the present study environment if the likely future cervical cancer epidemic is to be averted. The first rollout of HPV vaccination has just been commenced in Nigeria and this is expected to yield significant reductions in cervical cancer in few decades to come.

Young age at diagnosis of cancer among Africans is once more upheld in this study and this stretches the age bracket included in the analysis of age specific rates for each cancer type making most of them appear rare (3). Recent studies showed that cancer among adolescents and young adults is on the increase globally; although resource-limited communities are more affected. This could suggest disparities in risk exposures while the associated high mortality among the low economic group points to poor access to diagnostic services with most presenting at late stages (15, 29, 30). There is therefore the need to create awareness that would encourage early presentation, institute screening services for early detection, and enhance research to unravel familial or genetic risk factors prevalent in the population (15, 24).

The pattern of female cancers in this study is more consistent with other studies in Nigeria and globally being predominantly composed of breast, cervix, ovary, skin and uterine cancers, but that of males differed remarkably (2, 14). Haematolymphoid malignancies ranked high on the list of male cancers after prostate cancer in the index study but fifth in that documented by Jedy-Agba et al. in Abuja and Ibadan, Nigeria (14). Also, the finding of urinary bladder carcinomas in the first five cancers among males is similar to that reported among Americans by Siegel et al; in contrast, both lymphomas and urinary bladder cancers did not feature among top five male cancers globally in 2020 (2, 20). The northeast of Nigeria has a dual topography with rocks being very abundant as evidence of volcanic activity in time past that could emit ionizing radiation into the environment affecting the males more while they engaged in mining, arable and pastoral farming activities (11). Also, urinary bladder cancer could be due to a high burden of schistosomiasis as a remarkable proportion was squamous cell carcinoma, while the urothelial carcinoma histological subtype seen might suggest exposures to occupational risks and cigarette smoking (7). These all showed that epidemiological studies are needed in this region to first understand peculiarities of the disease among the people in order to design effective tailored control efforts for the region.

Given the burden of malignant diseases seen in this study, a high cancer mortality rate can be anticipated in the nearest future if preventive actions are not instituted immediately. This is more urgent because of the added severe lack of cancer care infrastructure in the region as highlighted in this study. Although survival data was not documented for this study, evaluation of cancer survival in Maiduguri, one of the sites included in this study, reported a mortality of about 85% for some cancers (31). Therefore, opportunities abound in SSA for multifaceted interventions that will help mitigate any catastrophic surge in cancer incidence and mortality rates in the next decade (7). Prevention should be prioritized as the malignancies with the most incident rates can be prevented through screening (32). Next, SSA countries should aim to retain the few available personnel to help sustain existing services and possibly reach the underserved populations. Thirdly, investments should be made to improve physical and human infrastructure in the hospitals for diagnosis and treatment of cancer patients on one hand, and to improve personnel capacity for research through training (3, 23–25). Partnership is required to achieve these, thus, Governments, Non-Governmental organizations, philanthropists, international partners, and Africa diaspora professionals have a role to play in birthing a SSA with extinct cancer mortality (3).

### Limitations

Data used in this study was hospital-based and consisted of only histologically diagnosed cases. This means that malignancies that are largely diagnosed clinically, such as liver and lung cancers may not be fairly represented. We recommend that the hospital cancer registries be expanded to population-based registries to cover cases diagnosed outside the tertiary hospitals. Also, clinically and radiologically diagnosed cancer cases should be included in the registry data and reflected in future studies to give a more reliable data estimates of the disease burden. Secondly, data was not received from two states in the region, therefore extrapolation of the findings of this study should be done with caution. Thirdly, we did not collect patient-level clinical and demographic data, nor did we assess for differences in survival. Future studies should evaluate these factors to test for their effects on cancer demographics in our population.

## Conclusion

Cancer incidence in this Nigerian community demonstrates a substantial burden which the existing cancer care system may not curtail. A multifactorial approach that includes prevention, screening/early detection, and effective treatment infrastructure is recommended to successfully address this disease. Relevant government institutions and donor agencies are enjoined to collaborate to bridge this gap. Further research efforts should aim at interventions targeting the top five cancers with the goal to reduce their incidences. Regular updates of this study data are also required to monitor the impact of control strategies.

## Data availability statement

The original contributions presented in the study are included in the article/**Supplementary Material**, further inquiries can be directed to the corresponding author.

## Ethics statement

The studies involving humans were approved by Research Ethics and Review Committee Federal Medical Centre Azare. The studies were conducted in accordance with the local legislation and institutional requirements. Written informed consent for participation was not required from the participants or the participants’ legal guardians/next of kin in accordance with the national legislation and institutional requirements.

## Author contributions

UE: Conceptualization, Data curation, Formal analysis, Methodology, Writing – original draft, Writing – review & editing. AL: Conceptualization, Data curation, Methodology, Writing – review & editing. MG: Conceptualization, Methodology, Supervision, Writing – review & editing. DS: Conceptualization, Data curation, Methodology, Validation, Writing – review & editing. DK: Conceptualization, Data curation, Methodology, Supervision, Writing – review & editing. AK: Conceptualization, Data curation, Methodology, Writing – review & editing. AA: Conceptualization, Data curation, Methodology, Writing – review & editing. AKM: Conceptualization, Data curation, Methodology, Writing – review & editing. OO: Methodology, Writing – review & editing. RD: Data curation, Methodology, Writing – review & editing. YA: Conceptualization, Methodology, Writing – review & editing. MA: Conceptualization, Methodology, Writing – review & editing. DB: Writing – review & editing. HU: Conceptualization, Writing – review & editing. AO: Conceptualization, Methodology, Writing – review & editing. MS: Conceptualization, Formal Analysis, Methodology, Writing – review & editing. SG: Conceptualization, Methodology, Supervision, Writing – review & editing. BA: Conceptualization, Methodology, Writing – review & editing.
