# Supplementary material for: Emerging cancer disease burden in a rural sub-Saharan African population: northeast Nigeria in focus
Source: Front Oncol. 2024 Jul 17;14:1380615. doi: 10.3389/fonc.2024.1380615 (PMC11288908; doi:10.3389/fonc.2024.1380615)
Supplement: Supplementary file 1 [file Table_1.docx]

Supplementary Table 1: Population of the states segregated by gender

| **States** | **Males** | **Females** | **Total** |
| --- | --- | --- | --- |
| **Adamawa** | 2,455,347 | 2,446,708 | 4,902,055 |
| **Bauchi** | 4,148,535 | 4,160,248 | 8,308,783 |
| **Borno** | 3,082,067 | 3,029,395 | 6,111,462 |
| **Gombe** | 2,052,241 | 1,907,881 | 3,960,122 |
| **Taraba** | 1,809,411 | 1,800,432 | 3,609,843 |
| **Yobe** | 1,854,775 | 1,794,832 | 3,649,607 |
| **Total** | **15,402,376** | **15,139,496** | **30,541,872** |
