# Supplementary material for: Emerging cancer disease burden in a rural sub-Saharan African population: northeast Nigeria in focus
Source: Front Oncol. 2024 Jul 17;14:1380615. doi: 10.3389/fonc.2024.1380615 (PMC11288908; doi:10.3389/fonc.2024.1380615)
Supplement: Supplementary file 2 [file Table_2.docx]

Supplementary Table 2 Age group by gender among the population

| **Age group** | **Male** | **Female** | **Total** |
| --- | --- | --- | --- |
| **0-4** | 2,598,386 | 2,524,709 | 5,123,095 |
| **5-9** | 2,263,043 | 2,224,031 | 4,487,074 |
| **10-14** | 2,095,811 | 2,068,171 | 4,163,982 |
| **15-19** | 1,738,137 | 1,666,537 | 3,404,674 |
| **20-24** | 1,481,597 | 1,370,235 | 2,851,832 |
| **25-29** | 1,151,264 | 1,024,174 | 2,175,438 |
| **30-34** | 907,821 | 846,637 | 1,754,458 |
| **35-39** | 717,379 | 868,588 | 1,585,967 |
| **40-44** | 587,508 | 770,255 | 1,357,763 |
| **45-49** | 499,035 | 556,704 | 1,055,739 |
| **50-54** | 413,026 | 410,737 | 823,763 |
| **55-59** | 330,471 | 295,256 | 625,727 |
| **60-64** | 251,224 | 208,314 | 459,538 |
| **65-69** | 166,936 | 134,947 | 301,883 |
| **70-74** | 104,360 | 83,810 | 188,170 |
| **75-79** | 56,227 | 48,582 | 104,809 |
| **80+** | 40,151 | 37,809 | 77,960 |
| **Total** | **15,402,376** | **15,139,496** | **30,541,872** |
